# Multi-spectroscopic, thermodynamic, and molecular docking/dynamic approaches for characterization of the binding interaction between calf thymus DNA and palbociclib

**DOI:** 10.1038/s41598-022-19015-9

**Published:** 2022-08-30

**Authors:** Galal Magdy, Moataz A. Shaldam, Fathalla Belal, Heba Elmansi

**Affiliations:** 1 Pharmaceutical Analytical Chemistry Department, Faculty of Pharmacy, Kafrelsheikh University, P.O. Box 33511, Kafrelsheikh, Egypt; 2 Pharmaceutical Chemistry Department, Faculty of Pharmacy, Kafrelsheikh University, P.O. Box 33511, Kafrelsheikh, Egypt; 3 Pharmaceutical Analytical Chemistry Department, Faculty of Pharmacy, Mansoura University, P.O. Box 35516, Mansoura, Egypt

**Keywords:** Analytical chemistry, Fluorescence spectroscopy, Spectrophotometry, Computational chemistry

## Abstract

Studying the binding interaction between biological macromolecules and small molecules has formed the core of different research aspects. The interaction of palbociclib with calf thymus DNA at simulated physiological conditions (pH 7.4) was studied using different approaches, including spectrophotometry, spectrofluorimetry, FT-IR spectroscopy, viscosity measurements, ionic strength measurements, thermodynamic, molecular dynamic simulation, and docking studies. The obtained findings showed an apparent binding interaction between palbociclib and calf thymus DNA. Groove binding mode was confirmed from the findings of competitive binding studies with ethidium bromide or rhodamine B, UV–Vis spectrophotometry, and viscosity assessment. The binding constant (*K*_*b*_) at 298 K calculated from the Benesi–Hildebrand equation was found to be 6.42 × 10^3^ M^−1^. The enthalpy and entropy changes (*∆H*^*0*^ and *∆S*^*0*^) were − 33.09 kJ mol^−1^ and 61.78 J mol^−1^ K^−1^, respectively, showing that hydrophobic and hydrogen bonds constitute the primary binding forces. As indicated by the molecular docking results, palbociclib fits into the AT-rich region of the B-DNA minor groove with four base pairs long binding site. The dynamic performance and stability of the formed complex were also evaluated using molecular dynamic simulation studies. The in vitro study of the intermolecular binding interaction of palbociclib with calf thymus DNA could guide future clinical and pharmacological studies for the rational drug scheming with enhanced or more selective activity and greater efficacy.

## Introduction

**Figure 1:**
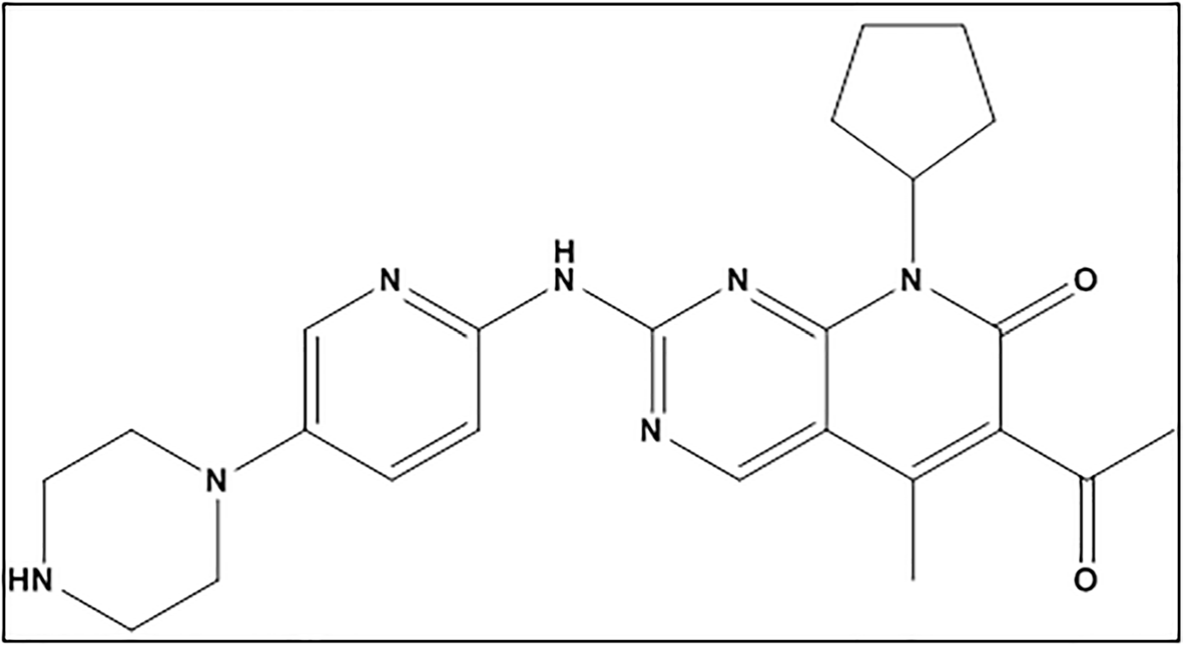
Chemical structure of PLB.

Palbociclib (PLB) is 6-acetyl-8-cyclopentyl-5-methyl-2-[(5-piperazin-1-ylpyridin-2-yl)amino]pyrido[2,3-d]pyrimidin-7-one^1^ (Fig. 1). It is an anti-cancer drug indicated for breast cancer under the trade name: Ibrance^®^ capsules. The capsules are present in different concentrations: 75, 100, and 125 mg as a free base^2^. PLB is used in combination with other drugs to treat hormone receptor-positive, advanced breast cancer (breast cancer that grows in response to hormones such as estrogen) or breast cancer that has spread to other parts of the body in women who have gone through menopause (the end of monthly menstrual periods). It is also used to treat breast cancer that has spread to other body regions in persons treated with antiestrogen drugs like tamoxifen. It has been considered by FDA to treat postmenopausal ER-positive/HER2-negative advanced breast cancer with letrozole as first-line therapy^3^. PLB belongs to the kinase inhibitor family of drugs. It functions by obstructing the action of the abnormal protein that signals cancer cells to multiply, which aids in preventing or slowing cancer cell spread^4^. As breast cancer is generally the second most common cancer and the most common cancer in women^5^, there is great importance for studying PLB molecular binding interaction with calf thymus DNA (CT-DNA) to get a clearer vision of its mechanism of action and pharmacological effect.

DNA draws out an essential role in life as it directs the biosynthesis of enzymes and proteins in a living cell by the replication and transcription of genetic information. It is, therefore, a vital carrier for this information. The interaction of small ligand molecules with DNA and other macromolecules gives an indication for drug design and synthesis or improvement of drugs with better selectivity and efficacy^6–20^.

Drugs can react with DNA through non-covalent or covalent bonds. Usually, the non-covalent pattern is predominant^21,22^. Different sites in the DNA molecule are susceptible to binding as follows: (1) in the minor groove, (2) in the major groove, (3) between two base pairs (full intercalation), (4) on the outside of the helix, and (5) electrostatic binding^23^. Usually, no single method can provide a complete vision for drug-DNA interaction. Therefore, it is essential to provide rapid, high throughput, continuous, and economical techniques for the assessment of the interaction of DNA with different drugs so as to help in drug discovery and approval processes.

From the literature, the in vitro binding interaction between PLB and CT-DNA was not reported, and the current work introduces the first study for exploring the binding interaction between PLB and CT-DNA, giving detailed information about the nature of this interaction including binding mode, binding constant, specific binding site, and interaction forces. Consequently, the current study aimed to conduct a comprehensive investigation of the interaction of PLB with CT-DNA employing a variety of approaches, including UV–visible spectrophotometry, spectrofluorimetry, FT-IR spectroscopy, viscosity measurements, and ionic strength measurements. Additionally, thermodynamic, molecular dynamic simulation, and molecular docking studies were also accomplished further to clarify binding mode, forces, and sites. Although PLB is an FDA-approved cyclin-dependent kinase inhibitor, the in vitro study of its intermolecular binding interaction with CT-DNA could provide guidance for future clinical and pharmacological studies for the rational drug scheming with enhanced or more selective activity and greater efficacy.

## Experimental

### Materials and chemicals

Palbociclib was kindly provided by Pfizer, Freiburg, Germany. PLB's stock solution was prepared in methanol with a final concentration of 1.0 × 10^−3^ M. CT-DNA, Tris–HCl, ethidium bromide (EB), and Rhodamine B (RB) were purchased from Sigma Aldrich (St. Louis, MO, USA). The chemicals used throughout this study were of analytical grade.

Tris–HCl buffer solution (0.05 M) was prepared in distilled water, and its pH was set at 7.4 in all experiments. Additionally, CT-DNA stock solution was obtained by dissolving in Tris–HCl buffer with frequent stirring to obtain a homogenous solution. CT-DNA solution's purity was assessed by computing the absorbance ratio of A_260_/A_280,_ which was found to be 1.903 (over 1.8), confirming that there is no protein contamination in CT-DNA^24–27^. The final concentration of CT-DNA solution was measured using the extinction coefficient of 6600 M^−1^ cm^−1^ of a single nucleotide at 260 nm (T = 298 K)^28–30^. The prepared CT-DNA solutions were kept at 4 °C and utilized within five days. Each of EB and RB (1.0 × 10^−3^ M) was prepared by dissolving in ethanol as they suffer from low water solubility and also stored at 4 °C.

### Instrumentation

- T80 + UV/VIS PC Spectrophotometer (PG Instruments Ltd., Woodway Lane, Wibtoft, England) with a 1.0 cm quartz cell was used for the spectrophotometric measurements.
- Agilent Technologies Cary Eclipse spectrofluorimeter with Xenon flash lamp (Santa Clara, CA 95051, USA) was used for the spectrofluorimetric measurements.
- Oswald Viscometer, at a controlled temperature of 298 K, was also used. The capillary's inner diameter was 0.57 mm.

### Study of PLB-CT-DNA interaction

#### Spectrophotometric measurements

The UV spectra of 8.25 × 10^−5^ M CT-DNA solutions were scanned from 200 to 400 nm upon adding increased concentrations of PLB in the range of (0–2.5 × 10^−5^ M) at four distinct temperatures (298, 303, 308, and 313 K) in order to estimate the binding constants and evaluate the temperature effect on the drug-CT-DNA interaction. The corresponding PLB solutions served as references. Furthermore, the absorbance value of a mixed solution of PLB (2.0 × 10^−5^ M) and CT-DNA (82.5 × 10^−5^ M) was examined at 298 K, varying the NaCl concentration from 0 to 0.07 M to evaluate the effect of ionic strength.

#### Spectrofluorimetric measurements

In the presence and absence of different PLB concentrations, fluorescence emission spectra were recorded for mixtures of CT-DNA (57.0 µM) with fluorescent probes; RB (4.0 µM) and EB (2.0 µM) corresponding to groove and intercalation binding probes, respectively. Excitation wavelengths for RB and EB were adjusted at 465 and 525 nm, with emission spectra obtained at 576 and 574 nm, respectively. All spectra were measured three times, and the average was calculated with a blank experiment conducted similarly.

#### Viscosity measurements

A viscosity study was performed at 298 K using increasing concentrations of PLB (0–2.5 × 10^−5^ M), whereas the CT-DNA concentration in Tris–HCl solution was maintained at 8.25 × 10^−5^ M. The flow times of the CT-DNA solutions were recorded using a digital stopwatch. An average of 3 determinations was recorded. The viscosity was calculated by referring to the formula (*η* = *(t *− *t*_*0*_*)/t*_*0*_), where (*t*) represents the measured flow times of CT-DNA-containing solutions and (*t*_*0*_) represents the flow time of Tris–HCl buffer alone. The average readings were utilized to determine the relative specific viscosity (*η/η*_*0*_)^1/3^, where *η* and *η*_*0*_ are the CT-DNA specific viscosities in the presence and absence of PLB, respectively^13,26^. The results were represented as (*η/η*_*0*_)^1/3^ against the binding ratio r (r = [PLB]/[CT-DNA]).

#### FT-IR spectroscopy

The Fourier Transform Infrared (FT-IR) spectra were recorded using ThermoFisher Scientific Nicolet-iS10 FT-IR Spectrometer, from Thermo Fisher Scientific (168 Third Avenue Waltham, MA, USA). It was equipped with a Ge/KBr beam splitter and a DTGS (deuterated triglycine sulfate) detector. Spectra were recorded as 100 scans with a resolution of 4 cm^−1^ from 4000 to 500 cm^−1^. PLB with CT-DNA samples were incubated for 2 h. The spectra of CT-DNA alone and PLB-CT-DNA complexes were recorded and subtracted from the background spectra^31,32^. A constant concentration of CT-DNA was used (14.0 mM) while a set of solutions containing different PLB concentrations were prepared, maintaining the ratios of PLB:CT-DNA at 1:10, 1:20, 1:50, and 1:100^32^.

#### Molecular docking

Docking studies were accomplished to explore the binding modes of PLB to three B-DNA sequences using AutoDock 4.2^33^. The 3D coordinates of three B-DNA sequences (1D29^34^, 3EY0^35^, and 1BNA^36^) were retrieved from Protein Data Bank (PDB) and were prepared using the Kollman approach^37^, which included adding partial atomic charges to DNA sequences. The PubChem Database ^1^ was used to obtain the PLB structure, and energy was minimized using Gaussian 03 software by density functional theory (DFT) at UB3lyp/6-311 + g(d) level till the eigenvalue of the Hessian matrix was positive. The genetic algorithm was utilized for energy evaluation as a search method with a population size of 100 and 2.5 million times. Table 1 specifies the grid box used for each DNA helix at a spacing point of 0.375. The 3D visualization and 2D schematic presentation for DNA-PLB complexes were generated by Chimera 1.13^38^ and LigPlot^+^ V1.4.5 software^39^, respectively.

**Table 1:** PDB entries and grid point data for the three DNA sequences used for the molecular docking study.

DNA (PDB ID)	Sequence	Grid points (coordinates/sizes)
3EY0	5′-(ATATATATAT)-3′	(16.394, 10.415, 90.220)/(80, 60, 60)
1D29	5′-(CGTGAATTCACG)-3′	(14.920, 20.905, 8.820)/(60, 60, 110)
1BNA	5′-(CGCGAATTCGCG)-3′	(14.780, 20.976, 8.807)/(60, 60, 110)

#### Molecular dynamic simulation

The DNA-PLB complex (3EY0) generated from the docking studies was constituted before simulation by adding hydrogen, optimizing, and solvating the complex using VMD 1.9^40^. DNA-PLB complex (3EY0) was placed in Periodic Boundary Conditions (PBC) water box and neutralized with NaCl (0.15 M). In this study, two molecular dynamic (MD) simulations were carried out, one for free DNA sequence (3EY0) and the other for PLB-associated DNA sequence. The MD simulation was carried out following the simulation parameters given in the literature^41^. Both MD simulations were executed for 50 ns using NAMD 2.13^42^ employing CHARMM36 force-field parameters^43^.

## Results and discussion

### Determination of binding mode of PLB with CT-DNA

#### Spectrophotometric measurements

**Figure 2:**
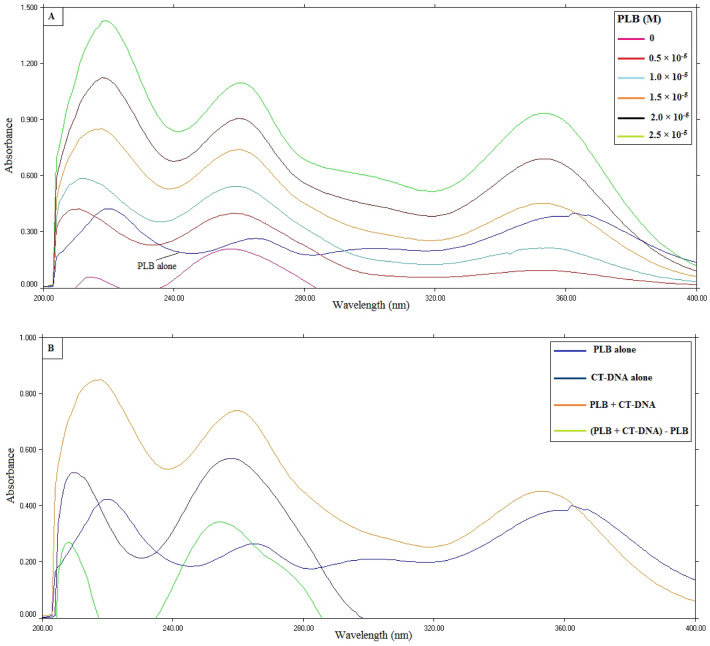
UV absorption spectra of (**A**) CT-DNA (8.25 × 10^−5^ M) titrated with increasing concentrations of PLB (0–2.5 × 10^−5^ M), (**B**) PLB alone, CT-DNA alone, PLB-CT-DNA complex, and the difference spectrum.

This approach is based on observing the alteration in the location and intensity of CT-DNA distinctive absorption bands at 260 nm, that are linked to the π–π* transition of DNA's base pairs^13,26^. It was observed that the intensity of the CT-DNA solution increased gradually upon the increment in the concentration of PLB, as illustrated in Fig. 2A, while the peak location remained almost unchanged. Accordingly, the binding manner of PLB with CT-DNA is suggested to be groove binding instead of intercalation, according to the shift rule for DNA's distinctive absorption peak^26,44,45^. The complex formation was further confirmed by non-overlapping of the absorption spectrum of PLB, CT-DNA, and the difference spectrum (Fig. 2B)^15,19^.

#### Viscosity measurements

**Figure 3:**
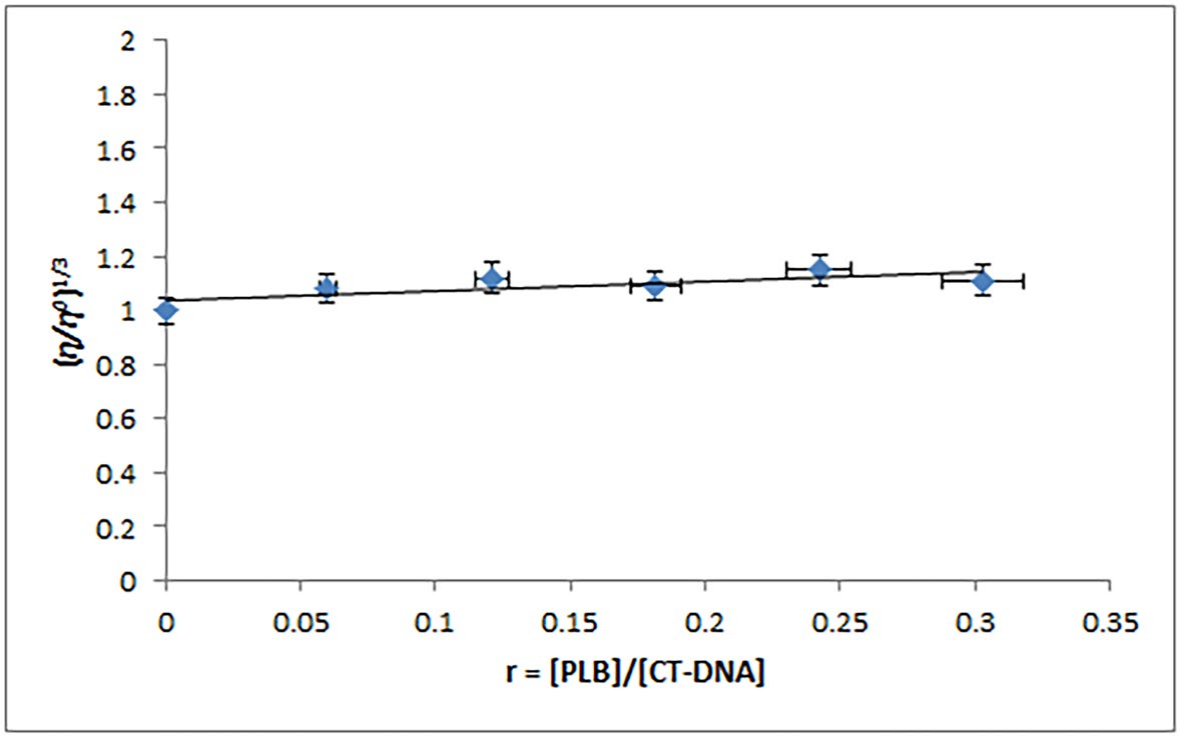
Influence of variable concentrations of PLB (0–25.0 μM) on the viscosity of CT-DNA (82.5 μM) in Tris–HCl buffer.

Viscosity measurements are highly sensitive to the variations in the DNA length^46^. The conventional intercalative binding mechanism has been observed to considerably alter the DNA solution viscosity as it needs a large and adequate space between consecutive base pairs to extend the double helix and provide accommodation for micro-molecules. Furthermore, a molecule's non-classical intercalation may cause the DNA helix to twist, lowering its length and viscosity. On the other hand, electrostatic and groove binding mechanisms have little to no effect on DNA viscosity^13,45–48^. The relative specific viscosity (*η/η*^*0*^)^1/3^ of CT-DNA was found to be practically constant, as shown in Fig. 3, confirming PLB groove binding with CT-DNA, which is compatible with the spectrophotometric study findings.

#### Assessment of competitive interactions

**Figure 4:**
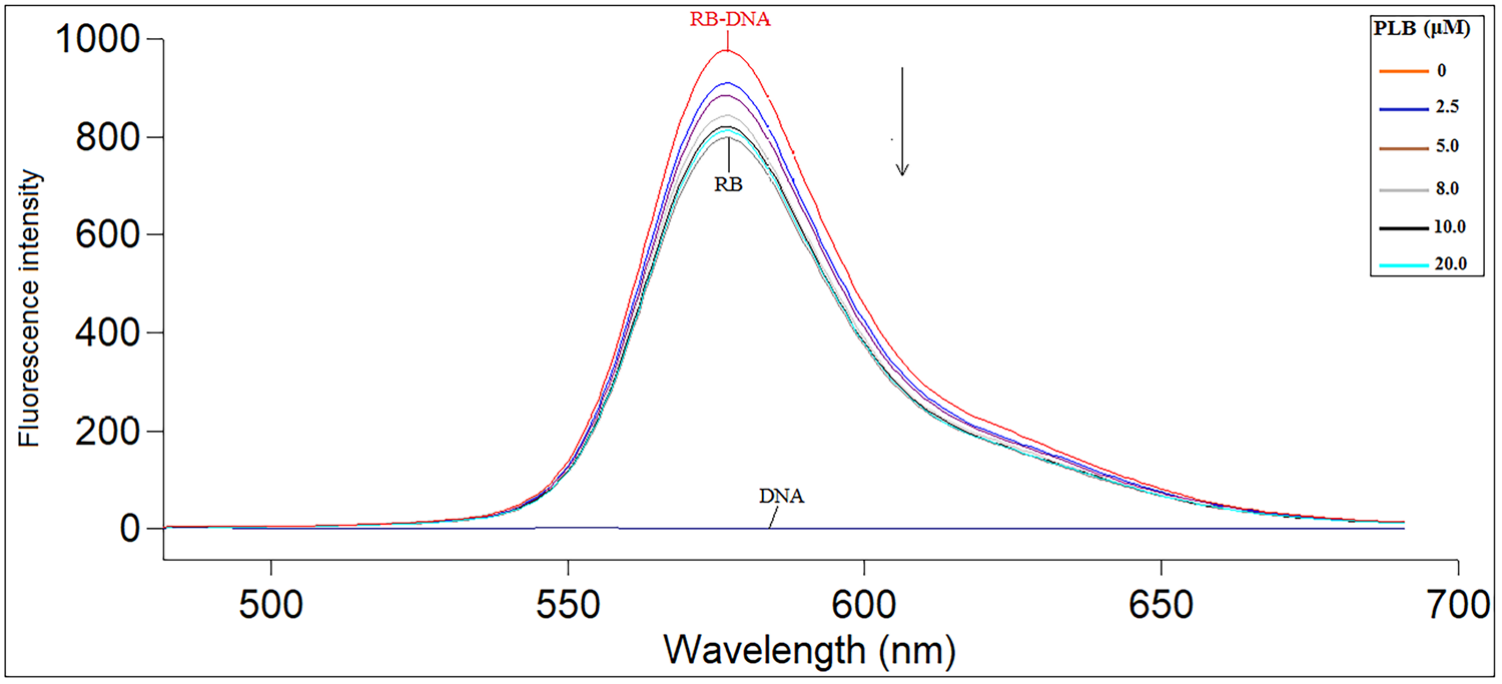
Fluorescence emission spectra of RB-CT-DNA complex at 298 K with or without PLB. C(CT-DNA): 57.0 μM; C(RB): 4.0 μM; C(PLB) (0–20.0 μM), (λ_ex._/λ_em._ = 465/576 nm).

The spectrofluorimetric competitive binding studies were carried out utilizing the fluorescent probes; RB and EB to learn more about how CT-DNA binds to PLB. Both EB and RB exhibit a considerable increase in fluorescence intensity upon binding to DNA^49,50^. It was found that RB attaches in the minor groove of DNA with a predilection for AT-rich regions, whereas EB attaches to DNA via intercalation, according to previously published studies^51,52^. After numerous trials, it was observed that adding PLB to the CT-DNA-EB complex did not almost affect the complex fluorescence intensity. The RB-CT-DNA complex fluorescence intensity reduced as PLB concentration increased (Fig. 4). Consequently, this proves that PLB and RB have a competitive binding relationship on CT-DNA, whereas PLB and EB do not have a competitive binding interaction. These results also support that PLB has a minor groove binding mechanism with CT-DNA instead of intercalation.

#### Effect of ionic strength

**Figure 5:**
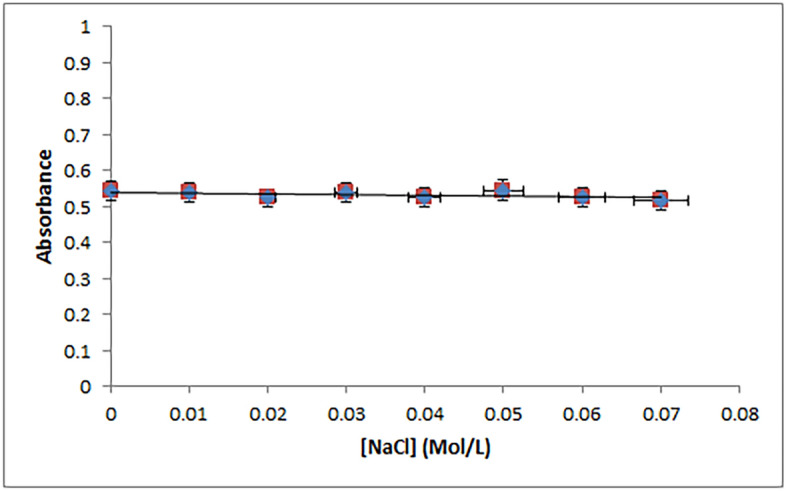
Influence of the ionic strength of NaCl on the absorbance of CT-DNA-PLB complex. Concentrations of PLB and CT-DNA were 2.0 × 10^−5^ M and 8.25 × 10^−5^ M, respectively. The concentrations of NaCl: 0, 0.01, 0.02, 0.03, 0.04, 0.05, 0.06, 0.07 M.

As is well known, the ionic strength of the reaction medium affects the intensity of electrostatic interaction significantly. Hence, under physiological settings, the electrostatic force is relatively weak and acts as an auxiliary force in the interaction between ligands and macromolecules^53^. The impact of changing the concentration of NaCl on the binding contact between CT-DNA and PLB was investigated to find out the probability of electrostatic interaction between them. The CT-DNA-PLB complex's absorbance value remained relatively constant as the concentration of NaCl was increased from 0 to 0.07 M, as presented in Fig. 5, indicating the absence of electrostatic interaction.

Overall, PLB and CT-DNA showed a minor groove binding interaction, according to the previously described experimental data.

### Evaluation of binding affinity between PLB and CT-DNA

Since the potency of a drug is directly connected to its binding affinity, studying the drug's binding affinity to a biomacromolecule is critical. The binding constant (*K*_*b*_) or dissociation constant (*K*_*d*_) could be used to determine the binding affinity. The *K*_*b*_ value of the 1:1 PLB-CT-DNA complex could be calculated by applying the Benesi-Hildebrand Eq. (1)^13,45^:

1 $$\frac{{A}_{0}}{A- {A}_{0}}= \frac{{\varepsilon }_{DNA}}{{\varepsilon }_{PLB{\text{-}}DNA}- {\varepsilon }_{DNA}}+ \frac{{\varepsilon }_{DNA}}{{\varepsilon }_{PLB{\text{-}}DNA}-{\varepsilon }_{DNA}} \times \frac{1}{{K}_{b } \cdot { C}_{PLB}}$$

where, *A* and *A*_*0*_ represent CT-DNA absorbance with and without PLB, respectively. ${\varepsilon }_{DNA}$ and ${\varepsilon }_{PLB{\text{-}}DNA}$ are CT-DNA and PLB-CT-DNA complex molar extinction coefficients, respectively. *C*_PLB_ is the PLB concentration.

**Figure 6:**
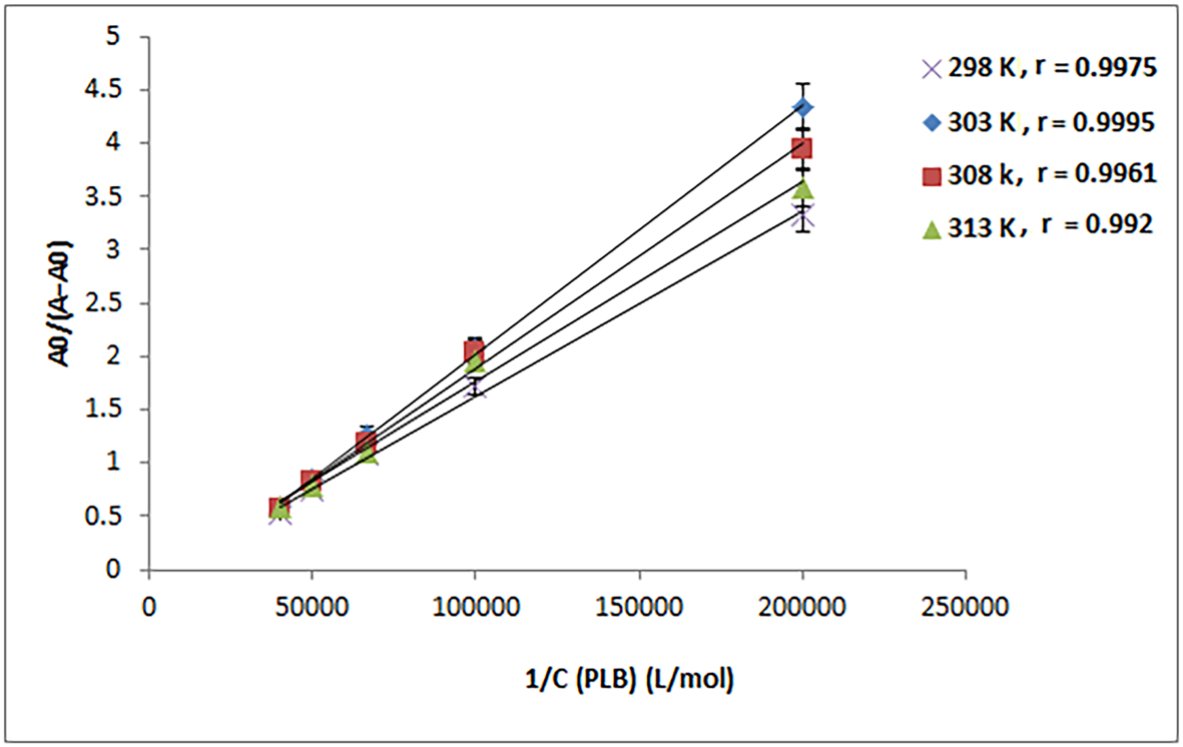
Plot of *A*_*0*_/(*A − A*_*0*_) versus 1/*C*_PLB_ at different temperature settings (*C*_DNA_ = 8.25 × 10^−5^ M), where r is the correlation coefficient.

As shown in Fig. 6, 1/*C*_PLB_ was plotted against *A*_*0*_/(*A *− *A*_*0*_) at each temperature (298, 303, 308, and 313 K). A linear correlation was found, indicating that the PLB-CT-DNA complex has a 1:1 stoichiometry. The *K*_*b*_ values for the PLB-CT-DNA complex were determined using Eq. (1), and the findings are abridged in Table 2. In the examined range of temperatures, the obtained *K*_*b*_ values were in the order of 10^3^ M^−1^, demonstrating that PLB has a moderate affinity for CT-DNA binding. Furthermore, the *K*_*b*_ values appear to be in the same range as groove binders^7,10,50,52,54^ and lower than those of classic intercalators, as the DNA-EB complex (*K*_*b*_ = 1.4 × 10^6^ M^−1^)^55,56^, confirming that the binding between PLB and CT-DNA is via a minor groove mechanism.

**Table 2:** Assessment of the binding constants (K_b_) at four different temperatures and thermodynamic parameters of the CT-DNA-PLB complex.

T (K)	K_b_ (M^−1^)	r	S.D	ΔH^0^ (kJ mol^−1^)	ΔS^0^ (J mol^−1^ K^−1^)	ΔG^0a^ (kJ mol^−1^)	ΔG^0b^ (kJ mol^−1^)
298	6.42 × 10^3^	0.9975	1.12	− 33.09	61.78	− 21.72	− 21.72
303	6.26 × 10^3^	0.9995	1.51			− 22.02	− 22.03
308	6.16 × 10^3^	0.9961	1.36			− 22.344	− 22.337
313	6.01 × 10^3^	0.992	1.23			− 22.642	− 22.646

^a^Δ*G*^*0*^ = *RT*ln*K*_*b*_*.*

^b^Δ*G*^*0*^ = Δ*H*^*0*^ − *T*Δ*S*^*0*^.

### Assessment of thermodynamic parameters and major interaction forces

Hydrogen bonding, electrostatic, hydrophobic, and van der Waals forces are four non-covalent binding forces that have been reported to contribute to the binding between biomacromolecules and small molecules^57^. Furthermore, the value and sign of entropy (ΔS^0^) and enthalpy (ΔH^0^) changes can be used to infer the type of binding forces. When both ΔS^0^ and ΔH^0^ are negative, it is typically assumed that the basic interaction forces are van der Waals force and/or hydrogen bonding. Hydrophobic interaction is the main force when both ΔS^0^ and ΔH^0^ are positive, but electrostatic interaction when ΔS^0^ is positive and ΔH^0^ is approximately zero^26,58^. Van't Hoff Eqs. (2, 3) were used to determine the thermodynamic parameters in the binding of PLB with CT-DNA, including Gibbs free energy change (ΔG^0^), ΔS^0^, and ΔH^0^^26^:

2 $$\mathrm{ln}{K}_{b }= - \frac{{\Delta H}^{^\circ }}{RT} + \frac{{\Delta S}^{^\circ }}{R}$$

3 $$\Delta {G}^{^\circ }= {\Delta H}^{^\circ }-T{\Delta S}^{^\circ }$$

where, R represents a gas constant.

**Figure 7:**
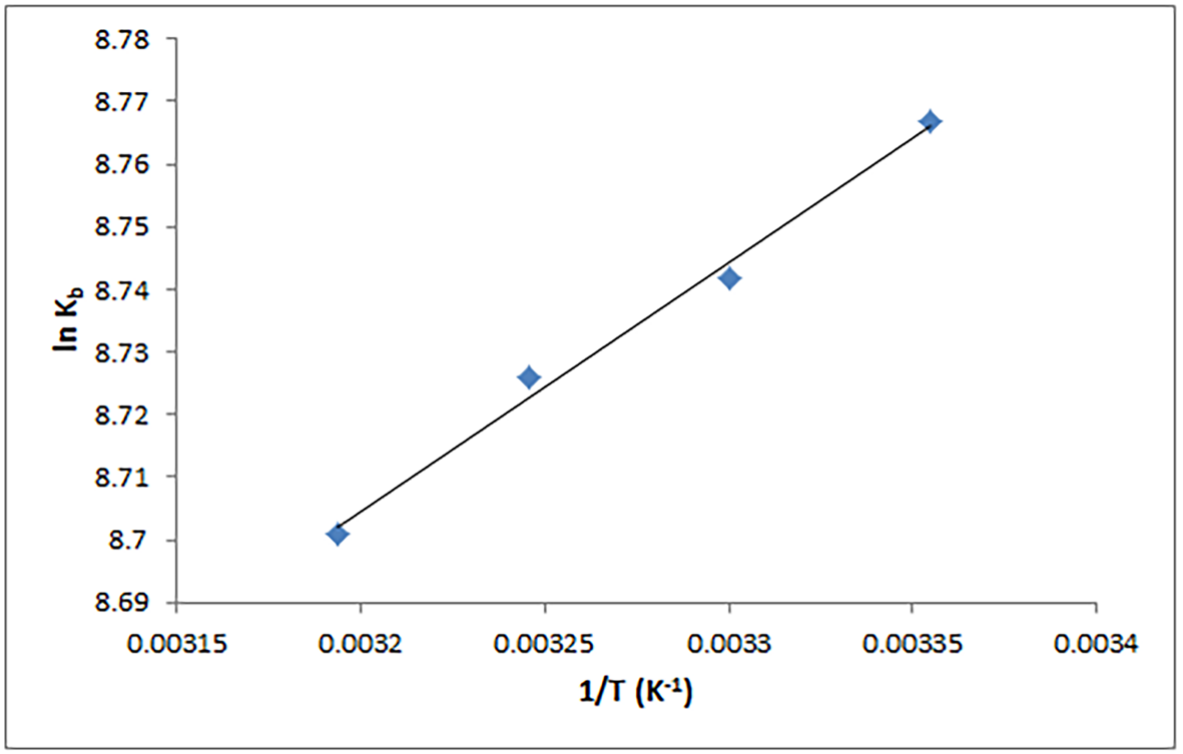
Van’t Hoff plot for the CT-DNA-PLB complex.

ΔH^0^ and ΔS^0^ values were determined using the slope and intercept of the van't Hoff plot of ln K_b_ against 1/T (Fig. 7) and are presented in Table 2. Because ΔG^0^ is less than zero, it is concluded that CT-DNA and PLB have a spontaneous binding relationship. The positive ΔS^0^ value is commonly because of the hydrophobic interaction. Furthermore, the negative value of ΔH^0^ is not an indicator of electrostatic interactions because ΔH^0^ is close to zero in the case of electrostatic interactions, implying that the negative ΔH^0^ value is likely due to hydrogen bonding interactions^7,50,58–60^. As a result, hydrophobic interactions and hydrogen bonding may be the primary binding forces for the PLB-CT-DNA interaction, as validated by molecular docking studies.

### Determination of conformational changes of CT-DNA

**Figure 8:**
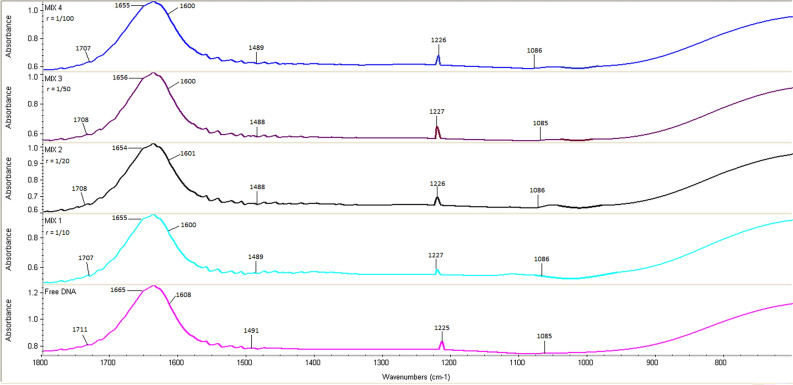
Stacked view of FT-IR spectra of free CT-DNA and PLB/CT-DNA ratios of 1/10, 1/20, 1/50, and 1/100 in the region of 1800–700 cm^−1^.

The conformational changes in the structure of DNA upon the binding interaction with the drugs or other small molecules could be assessed using FT-IR or circular dichroism (CD)^61–65^. Since FT-IR is non-destructive, requires little to no sample preparation, and provides information on all conformations present in the sample, it is an ideal technique for such studies^32^. The spectral characteristics of PLB-CT-DNA complexes are represented in Fig. 8. The region of interest lies in the spectral range 1800–700 cm^−1^, which is characterized by deoxyribose stretching of the DNA backbone, nitrogenous base ring vibrations, and PO_2_ stretching vibrations. The vibrational bands at 1711, 1665, 1608, and 1491 cm^−1^ are ascribed to the nitrogenous bases guanine (G), thymine (T), adenine (A), and cytosine (C), respectively. Bands at 1225 and 1085 cm^−1^ represent the symmetric and asymmetric phosphate vibrations, respectively^32,66^. These are the distinctive bands of pure CT-DNA examined in this study during the interaction of PLB with CT-DNA at different ratios, and the changes are illustrated in Fig. 8. After adding PLB to the CT-DNA solution, G, T, A, and C bands were observed to exhibit spectral shifts. However, the AT base pairs showed more shifting than GC base pairs which could be clarified by the fitting of PLB into the AT-rich region of the B-DNA minor groove, which is matched with the results of the molecular docking. No significant shifting is observed for phosphate vibrations. These results provide additional evidence that PLB binds to CT-DNA through direct interaction with CT-DNA nitrogenous bases (G, T, A, and C), while CT-DNA remains in the B-conformation^32,63^.

### Molecular modelling

**Figure 9:**
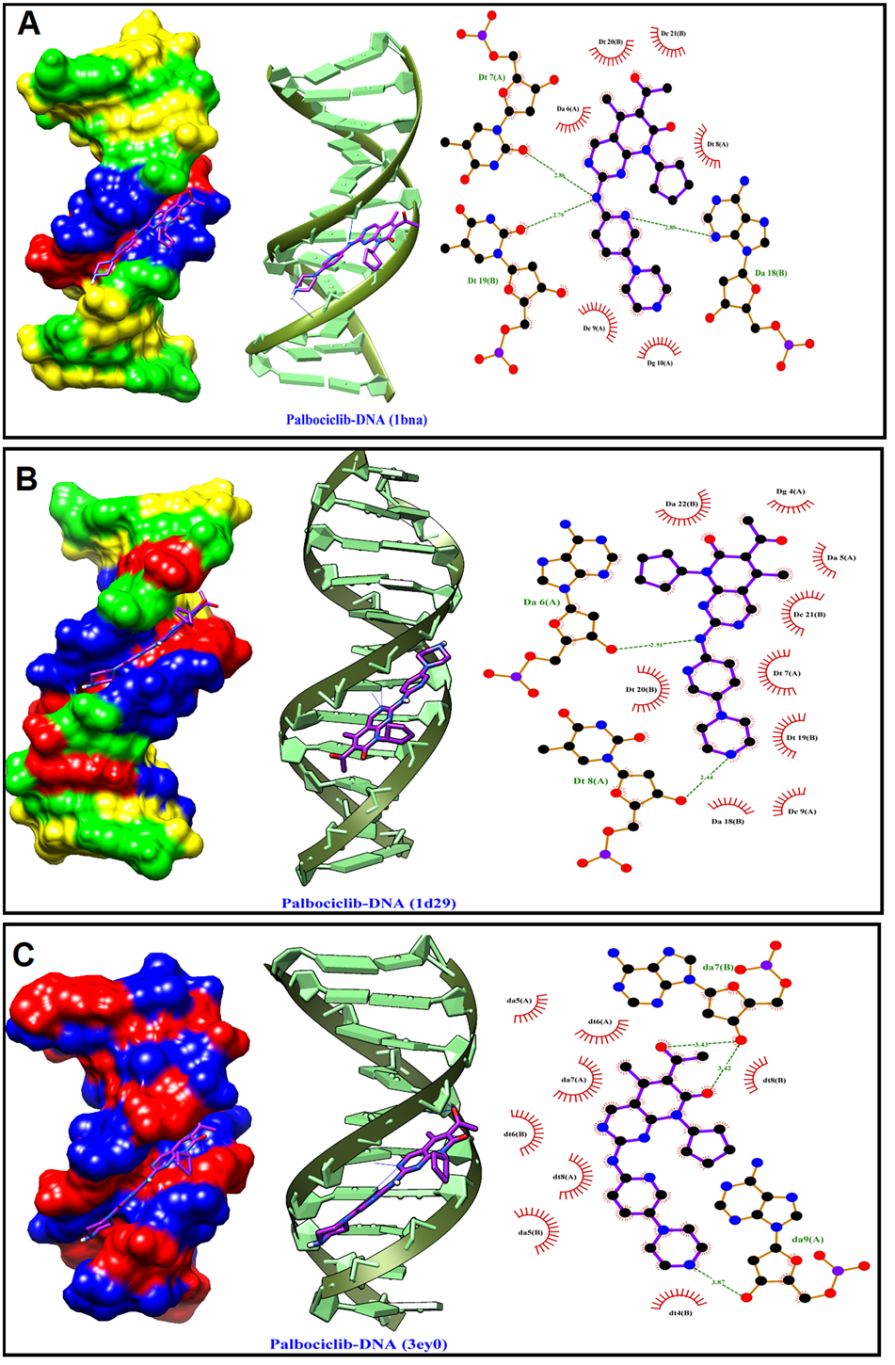
The lowest binding free energy pose for PLB on the three B-DNA fragments: Surface view (left) and 2D schematic view (right); deoxy cytosine (DC) is green, deoxy adenine (DA) is red, deoxy guanine (DG) is yellow, deoxy thymine (DT) is blue, cartoon presentation (right), H-bond interactions (green-dashed lines), hydrogen bond length (Å), and the hydrophobic interactions (red-rays).

Using AutoDock 4.2 software, a molecular docking study was performed to elucidate PLB's binding mechanism and elaborate the involved binding forces using three B-DNA sequences (1D29, 3EY0, 1BNA) with a well-studied structure which were used by many research groups for observing the location of the bound probe in CT-DNA^11,13,14,20,50,65,67^. PLB was manipulated as a flexible molecule with five active torsion positions when docked into the B-DNA fragments. With a four-base pair long binding site, PLB exhibited a preference for the AT-rich area of the B-DNA minor groove, as indicated by molecular docking studies (Fig. 9), and the predominant interaction was owing to hydrophobic interactions and hydrogen bonding, which agrees with competitive fluorescence probe assays and UV spectrophotometric measurements. The electrostatic energy (E3) could be excluded from PLB binding forces with DNA fragments when compared to the sum of energies created by other forces (E2), as shown in Table 3. Figure 9 also shows the hydrophobic interaction and hydrogen bonding in the three PLB-DNA complexes. PLB and the DNA basic groups formed three hydrogen bonds and multiple hydrophobic contacts in the PLB-DNA (1BNA and 3EY0) complex. Apart from the hydrophobic contacts in the minor groove area, PLB and DNA fragment (1D29) had two hydrogen bonding interactions. These findings were consistent with those of the thermodynamic study.

**Table 3:** The different binding energies (Kcal mol^−1^) participated in PLB-DNA complexes.

PLB-DNA complex	ΔG^a^	E_1_^b^	E_2_^c^	E_3_^d^
3EY0	− 12.88	− 14.37	− 11.49	− 2.88
1D29	− 11.62	− 13.11	− 10.49	− 2.62
1BNA	− 11.75	− 13.24	− 10.07	− 3.17

^a^ΔG: The binding free energy determined in water by the scoring function.

^b^E_1_: The intermolecular interaction energy, equals the sum of hydrogen bonding energy, van der Waals energy, electrostatic energy, and de-solvation free energy.

^c^E_2_: The sum of hydrogen bonding, van der Waals, and de-solvation free energies.

^d^E_3_: The electrostatic energy.

### Molecular dynamic simulation

**Figure 10:**
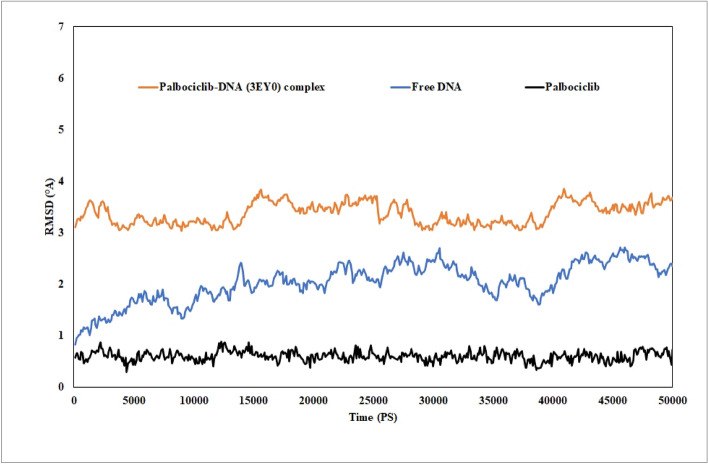
RMSD curves for the heavy atoms of free DNA (3EY0) (blue curve), its complex with PLB (orange curve), and free PLB (black curve) after 50 ns dynamic simulation experiments.

**Figure 11:**
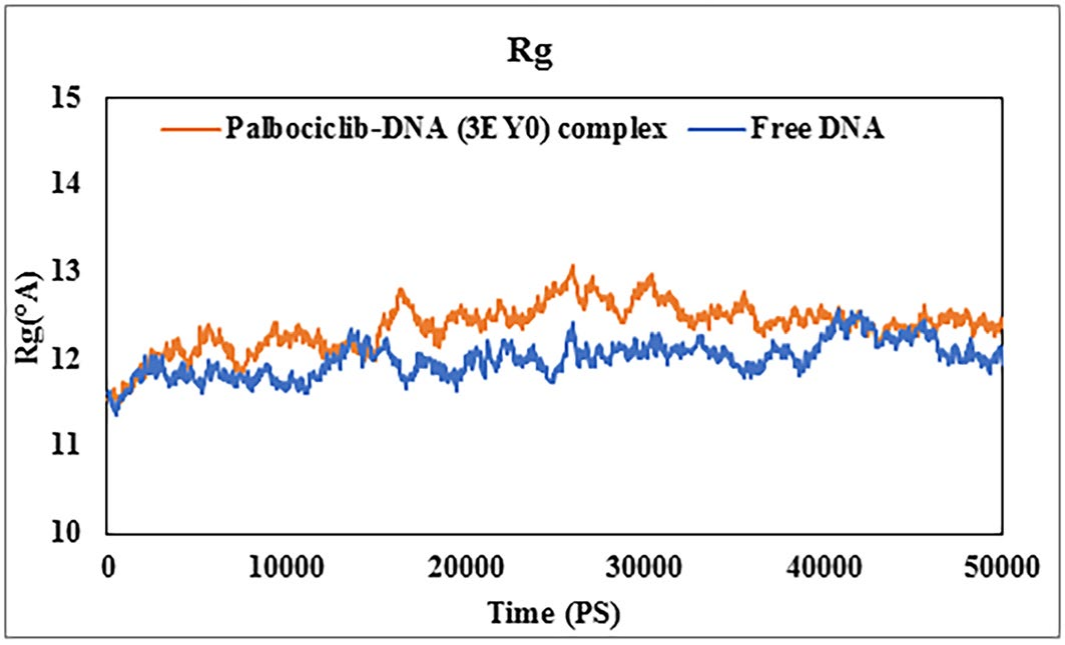
Radius of gyration (Rg) curves for free DNA (3EY0) (blue curve) and its complex with PLB (orange curve) after 50 ns dynamic simulation experiments.

**Figure 12:**
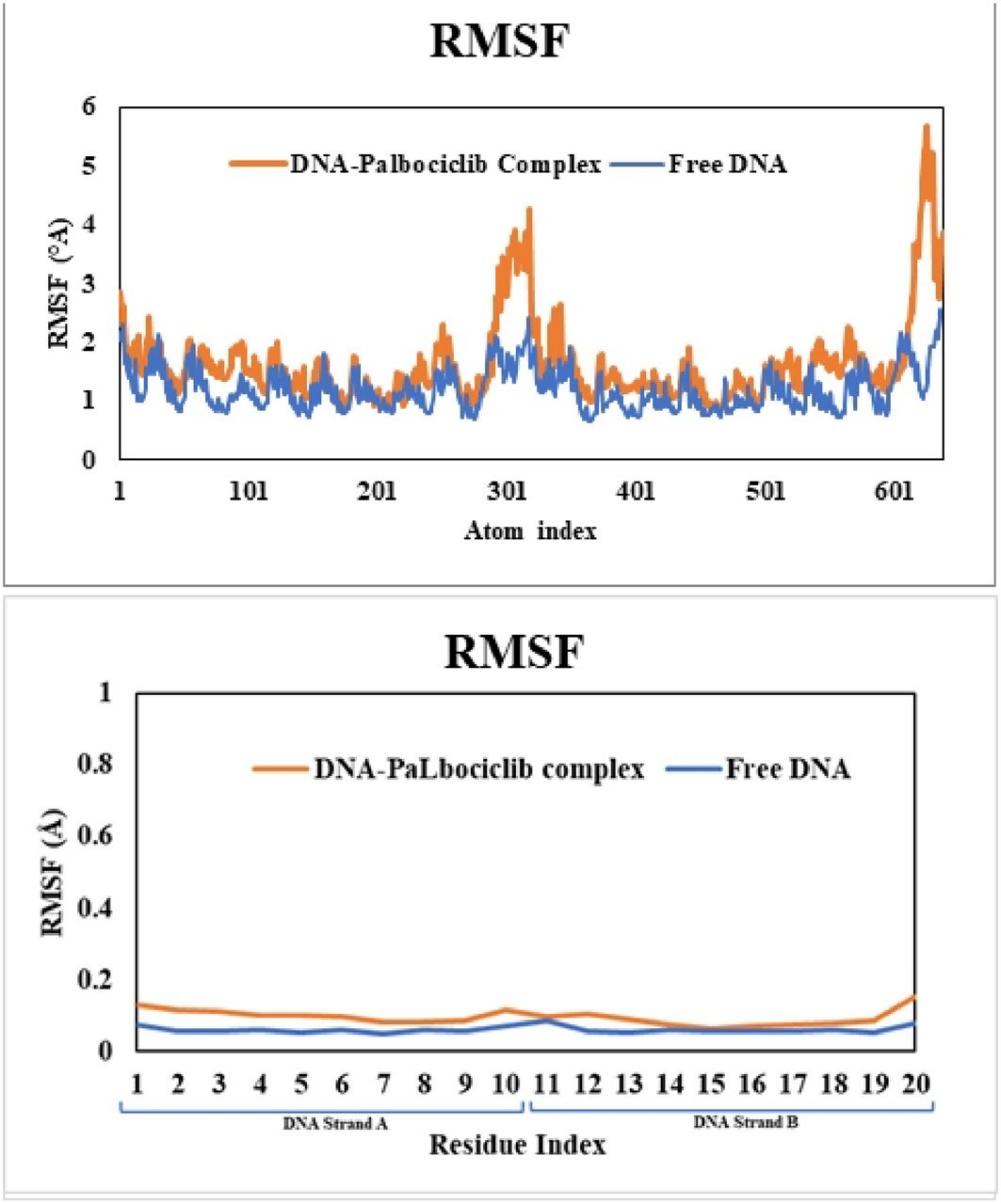
RMSF curves for free DNA (3EY0) (blue curve) and its complex with PLB (orange curve) after 50 ns dynamic simulation experiments per atom (Top) and DNA bases (Bottom) for strand A (atom 1–319, residue 1–10) and strand B (atom 320–638, residue 11–20).

An MD simulation study was conducted under physiologically mimic conditions to evaluate the stability and dynamic performance of the PLB-DNA (3EY0) complex, with the root mean square deviation (RMSD), a radius of gyration (Rg), and root mean square fluctuation (RMSF) computed by VMD and presented in Figs. 10, 11, and 12, respectively.

Following the initial 15 ns simulation, the RMSD curve (Fig. 10) for the heavy atoms of free DNA and the PLB-DNA complex revealed to some extent a plateau with a difference of less than 1 Angstrom, indicating that the system has been well equilibrated. Furthermore, the RMSD values of the free PLB heavy atoms showed high stability during the 50 ns simulation, demonstrating that the compound remained bound during the entire simulation time.

Rg is considered a measure of DNA compactness; accordingly, Rg measurements can provide information about changes in DNA structure and thus its structural stability^7,41,68^. Figure 11 demonstrates that during the MD simulation, the Rg values for free DNA and the DNA-PLB complex were nearly comparable with a difference of less than 1 Angstrom, indicating that when PLB attached to DNA did not induce any conformational changes, which was consistent with the results of the FT-IR spectroscopy.

**Figure 13:**
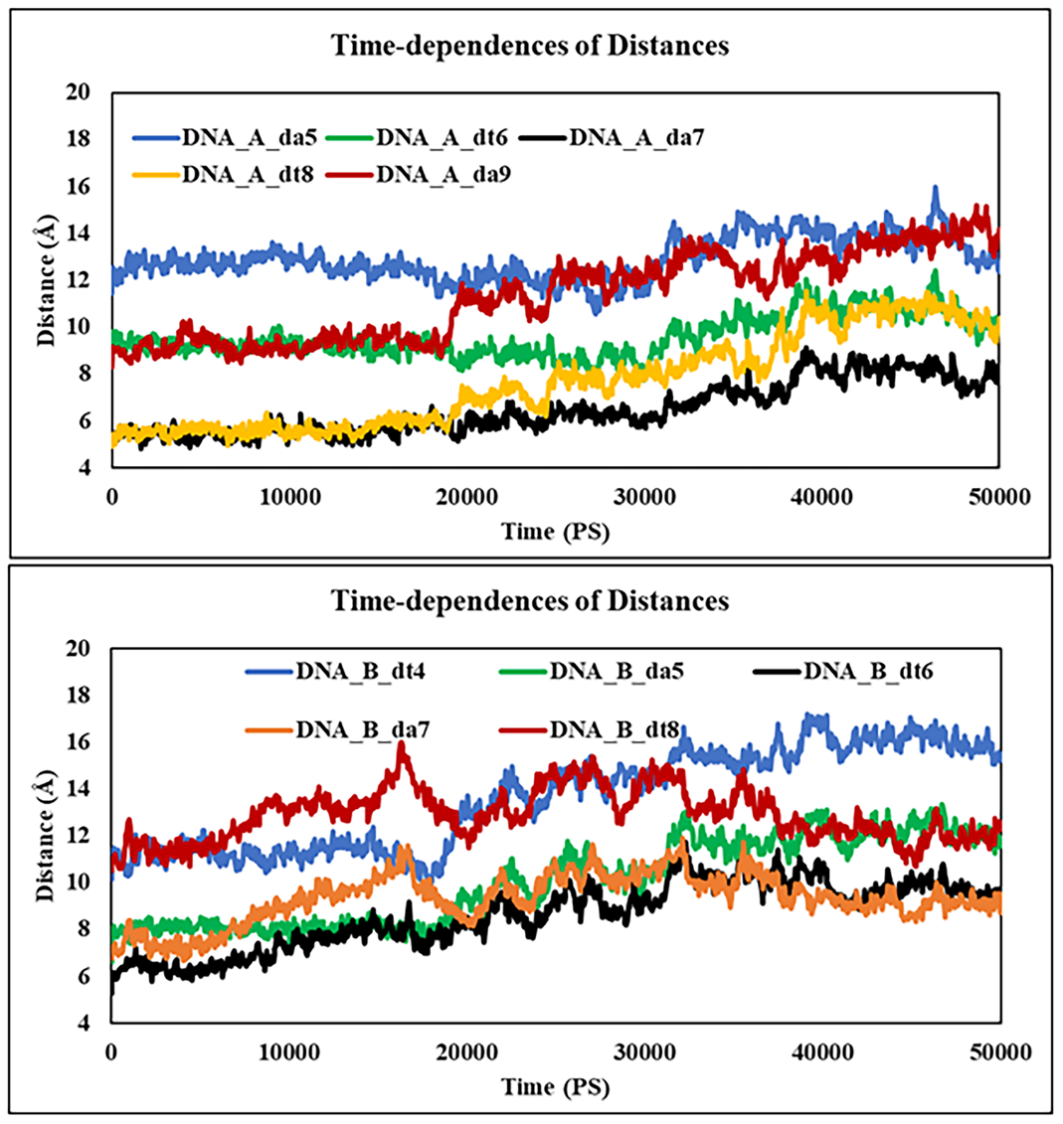
Time-dependence of distances for the center of masses of DNA bases with PLB showing the important interacting bases of strand A (Top) and strand B (Bottom).

The RMSF results (Fig. 12) showed that no significant fluctuation occurred in nearly all atoms of the bound DNA except for the DNA terminals that are remote from the PLB binding region. This is clearly observed from the RMSF values per DNA bases, where the bound DNA values showed nearly typical values of free DNA, and this supports the stability of the formed complex. Moreover, the distance changes over the 50 ns simulation time showed high stability as observed in small changes in the distances of the interacting bases with PLB showing bases thymine(A)6, adenine(A)7 thymine(A)8, adenine(A)9, adenine(B)5, thymine(B)6, adenine(B)7, and thymine(B)8 which were in close contact to the ligand (Fig. 13). Overall, the MD simulation results supported the experimental and docking data, confirming that PLB bound to CT-DNA in a stable manner.

## Conclusion

The in vitro molecular binding experiments have a substantial advantage that allows a precise assessment of the interaction between small molecules and DNA. The current work introduces the first study of the binding interaction of PLB with CT-DNA by applying several spectroscopic and in silico approaches. Through the examination of UV–Vis absorption and viscosity measurements, the complex formation between PLB and CT-DNA has been confirmed via a minor groove binding manner. The calculated binding constant was in the range of 10^3^ M^−1^, which is in the same range of familiar groove binders, confirming the moderate binding affinity. Competitive spectrofluorimetric displacement studies using RB and EB revealed that PLB interacts with CT-DNA through groove binding rather than intercalation. The conformational changes of CT-DNA structure in the binding interaction with PLB were also studied using FT-IR spectroscopy. Evaluation of the thermodynamic parameters revealed that PLB's binding to CT-DNA was spontaneous, and hydrogen bonding and hydrophobic interactions were the primary binding forces that stabilized the CT-DNA-PLB complex. The experimental results were further confirmed by the molecular docking and molecular dynamic simulation studies. Overall, this research offered detailed information about the nature of this interaction, including binding mode, binding constant, specific binding site, and interaction forces which are worthful for the rational drug scheming with enhanced or more selective activity and greater efficacy.

## Data Availability

The datasets generated and/or analyzed during the current study are available from the corresponding author on reasonable request.
